# Etanercept ameliorates psoriasis progression through regulating high mobility group box 1 pathway

**DOI:** 10.1111/srt.13329

**Published:** 2023-04-18

**Authors:** Shu Li, Guangli Li, Xiaoyan Li, Fan Wu, Ling Li

**Affiliations:** ^1^ Department of Dermatology Taizhou People's Hospital Taizhou P. R. China; ^2^ Internal Medicine Department Fushun Maternal and Child Health Hospital Fushun P. R. China; ^3^ Department of Dermatology Lianshui County People's Hospital Huai 'an P. R. China

**Keywords:** etanercept, HaCaT cells, HMGB1 signaling pathway, inflammation, psoriasis

## Abstract

**Background:**

As a common skin disease, psoriasis is related to inflammation and immune response. Due to the frequent recurrence of psoriasis, the treatment of psoriasis remains a clinical challenge. As an effective tumor necrosis factor‐alpha (TNF‐α) inhibitor, etanercept has been used for the treatment of psoriasis. However, some patients with psoriasis have no response to etanercept or discontinue treatment. To improve the therapeutic effect of etanercept, searching the potential biomarkers and investigating the related mechanisms of etanercept in the treatment of psoriasis are vital.

**Materials and methods:**

We stimulated HaCaT cells with lipopolysaccharide (LPS) to generate cellular psoriatic changes and established an imiquimod (IMQ)‐induced psoriasis‐like mouse model, and then used an etanercept to treat cell and mouse model.

**Results:**

Etanercept alleviated IMQ‐induced pathological changes and inflammation, and it also decreased the protein expression of high mobility group box 1 (HMGB1), receptor for advanced glycation end‐products, and toll‐like receptor 4. Moreover, the results of in vitro experiments showed that etanercept inhibited proliferation and inflammation, and promoted cell cycle arrest and apoptosis in LPS‐treated HaCaT cells. Knockdown of HMGB1 further enhanced the inhibitory effects of etanercept on LPS‐treated HaCaT cell viability and inflammation, while overexpression of HMGB1 notably reversed the inhibitory effects of etanercept on LPS‐induced HaCaT cell viability and inflammation.

**Conclusion:**

Etanercept inhibited proliferation and inflammation and promoted cell cycle arrest and apoptosis in LPS‐induced HaCaT cells, and etanercept ameliorated inflammation in a psoriasis‐like mouse model.

**Abbreviations**
CCK‐8: Cell Counting Kit‐8
CST: cell signaling technology
EdU: 5‐ethynyl‐2′‐deoxyuridine
ELISA: enzyme‐linked immunosorbent assay
H&E: hematoxylin and eosin
HMGB1: high mobility group box 1
IL: interleukin
IMQ: imiquimod
LPS: lipopolysaccharide
MTX: methotrexate
NF‐κB: nuclear factor kappa B
PBS: phosphate‐buffered saline
PD: pharmacodynamic
PI: propidium iodide
PK: pharmacokinetics
RAGE: receptor for advanced glycation end products
RT‐qPCR: reverse transcription‐quantitative polymerase chain reaction
TLR2/4: toll‐like receptor 2/4
TNF‐α: tumor necrosis factor‐alpha

## 1 INTRODUCTION

Psoriasis is a chronic autoimmune disease, which affects 1%–3% of the population worldwide.^1^ Psoriasis is clinically manifested as thickening and scaling of skin lesions, which are characterized by epidermal hyperplasia, aberrant differentiation of keratinocytes, and immune cell infiltration.^2^ Psoriasis has a high incidence rate and is prone to relapse, and is usually accompanied by complications, including type 2 diabetes, cardiovascular disease, and depression.^3^, ^4^ Notably, psoriasis has a substantial negative impact on the daily quality of life of the patients, such as limitations on clothing, relationship problems, and fertility problems.^5^ However, the exact pathogenesis of psoriasis has not been fully understood, and there is often no complete cure without a complete treatment plan.

The pathogenesis of psoriasis is complex and uncertain and involves heredity, immune regulation disorders, environmental factors, and so on.^6^ According to the evidence from molecule and clinic, neutrophils, T cells, and inflammatory cytokines (such as tumor necrosis factor‐alpha [TNF‐α], interleukin [IL]‐23, IL‐6, and IL‐8) are the key mediators for psoriasis progression.^7^, ^8^, ^9^ Compared with the uninvolved skin of patients with psoriasis and healthy individuals, TNF levels in psoriasis tissue are elevated, suggesting that blocking TNF activity contributes to treating psoriasis.^10^ At present, some patients with psoriasis are incurable, but it can be controlled with medication.^11^ Etanercept, a dimeric fusion protein, is considered to be an effective TNF‐α inhibitor.^12^, ^13^ Clinically, etanercept can be used to treat rheumatoid arthritis, ankylosing spondylitis, and psoriasis.^14^, ^15^, ^16^, ^17^, ^18^, ^19^ For patients with moderate to severe plaque psoriasis, etanercept is an important therapeutic option.^19^ In the USA, 50 mg twice weekly (BIW) for 3 months followed by a maintenance dosage of 50 mg/week is the recommended dosage of etanercept for patients with psoriasis.^18^ Nestorov et al. made population pharmacokinetics (PK)‐pharmacodynamic (PD) analysis in psoriasis, PK data were from three clinical studies with doses of 25 mg once weekly (QW), 25 mg BIW, and 50 mg BIW, and the results showed that the population means of the apparent steady‐state clearance in males (0.129 L/h) and females (0.148 L/h) was different and the population means of the apparent volume of distribution also varied with time (during week 1, 16.11; during weeks 2–4, 20.01; after week 4, 22.51).^20^

High mobility group box 1 (HMGB1), also known as amphoterin, is a ubiquitous nuclear protein in all cell types.^21^, ^22^ After cell damage, HMGB1 can be released into the extracellular milieu, where it functions as a damage‐associated molecular pattern molecule.^23^ HMGB1 can activate the nuclear factor kappa B (NF‐κB) pathway and subsequently induce pro‐inflammatory cytokines, such as IL‐1 and TNF‐α.^24^ HMGB1 is reported to modulate cytokine‐like activity by interacting with cellular receptors, such as receptor for advanced glycation end products (RAGE) and toll‐like receptor 2/4 (TLR2/4).^25^ Moreover, extracellular HMGB1 enhances the inflammatory response by binding to other pro‐inflammatory mediators, such as nucleic acids, lipopolysaccharide (LPS), IL‐1α, and β.^26^ Recently, a large number of studies have revealed that HMGB1 plays important role in many types of inflammatory and autoimmune diseases.^27^, ^28^, ^29^ It has been found that the level of HMGB1 in the serum of patients with psoriasis was increased, and HMGB1 expression was detected in the cytoplasm of psoriatic lesions.^21^, ^30^ These studies suggest that HMGB1 may play a key role in the pathogenesis of psoriasis. However, detailed studies investigating the role of etanercept and HMGB1 in psoriasis are lacking.

In this research, we hypothesized that etanercept regulated the HMGB1 pathway to influence psoriasis progression. Psoriasis‐like lesions induced by imiquimod (IMQ) are similar to human psoriasis in phenotype and histological characteristics.^31^ The aim of this study was to evaluate the influences of etanercept on LPS‐induced human keratinocyte HaCaT cells and IMQ‐induced psoriasis‐like mouse model. Moreover, the possible molecular mechanism related to the HMGB1 signaling pathway was also analyzed. Our findings may be helpful for understanding the therapeutic effects of etanercept in psoriasis.

## 2 MATERIALS AND METHODS

### 2.1 Cell culture

HaCaT cells were obtained from the Guangzhou Cellcook Biotech Co., Ltd (China) and cultured in Dulbecco's modified Eagle's medium (Gibco, Carlsbad, CA, USA) supplemented with 10% fetal bovine serum (Hyclone, Logan, UT, USA) and 1% penicillin and streptomycin (Sigma‐Aldrich; Merck KGaA, Darmstadt, Germany). The cells were cultured at 37˚C in a humidified incubator (Thermo Fisher Scientific, Waltham, MA, USA) containing 5% CO_2_. The cells from passage 3 were used in the follow‐up experiments.

### 2.2 Cell treatment and transfection

HaCaT cells were starved in a serum‐free medium for 12 h before treatment and transfection. Next, HaCaT cells were exposed to different doses of etanercept (0, 5, 10, 20, 40, and 80 μg/ml; Shanghai Sunshine Guojian Pharmaceutical Co., Ltd., China) for 24 h at 37˚C in 5% CO_2_. To further examine the effects of etanercept in HaCaT cells, we constructed hyperproliferative psoriatic keratinocytes using LPS.^32^ HaCaT cells were divided into Control (without treatment), LPS (HaCaT cells at a density of 1 × 10^5^ cells/ml were treated with 10 μg/ml LPS for 24 h), LPS+5 μg/ml Etanercept (HaCaT cells at a density of 1 × 10^5^ cells/ml were treated with 5 μg/ml etanercept for 6 h before LPS treatment), LPS + 40 μg/ml Etanercept (HaCaT cells at a density of 1 × 10^5^ cells/ml were treated with 40 μg/ml etanercept for 6 h before LPS treatment), LPS+Etanercept+si‐HMGB1 (HaCaT cells at a density of 1 × 10^5^ cells/ml were transfected with siRNA‐HMGB1 and treated with 40 μg/ml etanercept for 6 h before LPS treatment), and LPS+Etanercept+HMGB1 (HaCaT cells at a density of 1 × 10^5^ cells/ml were transfected with pcDNA3.1‐HMGB1 and treated with 40 μg/ml etanercept for 6 h before LPS treatment). The plasmid pcDNA3.1‐HMGB1 and siRNA‐HMGB1 were purchased from GenePharma (Shanghai, China). Lipofectamine™ 3000 reagent (Invitrogen, Carlsbad, CA, USA) was used for transfection.

### 2.3 Cell viability assay

In accordance with the manufacturer's instructions, Cell Counting Kit‐8 (CCK‐8) (Dojindo, Kumamoto, Japan) was used to measure cell viability. In brief, HaCaT cells (1 × 10^5^) were seeded into 96‐well plates and then treated with indicated chemicals for 24 h or 48 h. Next, cells were incubated with 10 μl CCK‐8 solution for 2 h at 37°C. Finally, the absorbance was read at 450 nm by a microplate reader (Thermo Fisher Scientific).^33^

### 2.4 5‐ethynyl‐2′‐deoxyuridine assay

Cell proliferation was detected by 5‐ethynyl‐2′‐deoxyuridine (EdU) assay using the Cell‐Light EdU Apollo567 In Vitro Imaging Kit (Ribobio Co., Guangzhou, China). Briefly, HaCaT cells (1×10^4^ cells) were seeded in the 96‐well plates. After the indicated treatment, cells were incubated with 100 μl EdU (50 μM) for 2 h at 37°C. Next, cells were fixed with 4% paraformaldehyde (Sigma‐Aldrich) for 15 min, permeabilized with 0.5% Triton X‐100 for 20 min, and then incubated with EdU solution and Hoechst 33342 (5 μg/ml) for 30 min in dark. Finally, the proportion of EdU‐positive cells was counted under a fluorescent microscope (Nikon, Japan).^34^

### 2.5 Cell cycle analysis

Cell cycle analysis was performed with cell cycle assay kit reagents (Sigma‐Aldrich) by flow cytometry. Briefly, HaCaT cells (1×10^6^ cells/well) were seeded in the 6‐well plates. After the indicated treatment, cells were washed with cold phosphate‐buffered saline (PBS, Sigma‐Aldrich), and then fixed in 75% ethanol at 4^∘^C. On the next day, cells were washed with PBS twice and then incubated with propidium iodide (PI) /Rnase A in the dark at 37°C for 30 min. Cell cycle phases were analyzed by flow cytometry (BD Biosciences, Franklin Lakes, NJ, USA).^35^

### 2.6 Apoptosis analysis

HaCaT cell apoptosis was measured with Annexin V‐FITC/PI apoptosis detection kit (BD Biosciences). In short, HaCaT cells (5×10^4^ cells/well) were seeded in the 6‐well plates. After the indicated treatment, cells were harvested, washed with PBS, and then incubated with annexin‐V fluorescein isothiocyanate (AnnexinV‐FITC) and PI for 20 min at room temperature in the dark. Subsequently, the percentage of cell apoptosis was detected by flow cytometry (BD Biosciences).^36^ Apoptosis rate refers to the sum of early cell apoptosis rate and late cell apoptosis rate. Gating strategy for apoptotic cells presented by early cell apoptosis (Annexin V+PI−), late cell apoptosis (Annexin V+PI+), or necrosis (Annexin V−PI+).

### 2.7 Western blot analysis

The total proteins were extracted from tissues and HaCaT cells using RIPA lysis buffer (Beyotime Biotechnology, China). Protein concentration was determined using a bicinchoninic acid assay kit (Thermo Fisher Scientific). Equal amounts of proteins (15 μg) were separated on 10% sodium dodecyl sulfate‐polyacrylamide gel electrophoresis and then transferred onto polyvinylidene fluoride membranes (Thermo Fisher Scientific). After blocking with 5% non‐fat milk for 2 h at 37°C, the membranes were incubated with primary antibodies [rabbit monoclonal anti‐Cyclin A, cat. no. 91500, 1: 1000 dilution, Cell Signaling Technology (CST); rabbit monoclonal anti‐Cyclin D1, cat. no. 55506, 1: 1000 dilution, CST; rabbit monoclonal anti‐cleaved caspase‐3, cat. no. 9654, 1: 1000 dilution, CST; rabbit monoclonal anti‐cleaved caspase‐9, cat. no. 7237, 1: 1000 dilution, CST; rabbit monoclonal anti‐HMGB1, cat. no. 6893, 1: 1000 dilution, CST; rabbit polyclonal anti‐p‐p65, cat. no. 3031, 1: 1000 dilution, CST; mouse monoclonal anti‐IκBα, cat. no. 4814, 1: 1000 dilution, CST; rabbit monoclonal anti‐glyceraldehyde‐3‐phosphatedehydrogenase, cat. no. 5174, 1: 1000 dilution, CST; mouse monoclonal anti‐β‐actin, cat. no. 93473, 1: 1000 dilution, CST; rabbit polyclonal anti‐RAGE, cat. no. ab37647, 1: 1000 dilution, Abcam; mouse monoclonal anti‐TLR4, cat. no. sc‐293072, 1: 1000 dilution, Santa Cruz Biotechnology.] at 4°C overnight, and then incubated with horseradish peroxidase‐conjugated secondary antibodies (goat anti‐rabbit IgG, cat. no. sc‐2004, 1:5000 dilution, Santa Cruz Biotechnology; goat anti‐mouse IgG, cat. no. ab6789, 1:5000 dilution, Abcam) for 2 h at 37°C. Finally, the protein bands were visualized using enhanced chemiluminescence (Beyotime) and analyzed by ImageJ software (NIH, Bethesda, MD, USA).^37^

### 2.8 Reverse transcription‐quantitative polymerase chain reaction

Total RNA was extracted from tissues and HaCaT cells using Trizol reagent (Invitrogen), and then RNA was reversely transcribed into complementary DNA with Transcriptor First Strand cDNA Synthesis Kit (Roche, Indianapolis, IN, USA). Reverse transcription‐quantitative polymerase chain reaction (RT‐qPCR) was performed with the QuantiTect SYBR Green RT‐qPCR Kit (Qiagen, Hilden, Germany) on Applied Biosystems 7300 Fast Dx RealTime PCR Detection System (Thermo Fisher Scientific). The comparative Ct method was used to quantify target gene (IL‐8, IL‐6, and TNF‐α) expression. The relative mRNA expression of IL‐8, IL‐6, and TNF‐α was normalized to β‐actin (internal control) using the 2^−ΔΔCt^ method.^38^ ΔΔCt = (Ct_target gene—_Ct_internal control_) _experimental group_—(Ct_target gene_—Ct_internal control_) _normal control group_.

### 2.9 Enzyme‐linked immunosorbent assay

After the indicated treatment, cell culture supernatants from HaCaT cells were collected and stored at –20˚C for cytokine measurements. The levels of IL‐8, IL‐6, and TNF‐α were measured using their commercial enzyme‐linked immunosorbent assay (ELISA) kits (Bioswamp Life Science Lab, Wuhan, China) according to the manufacturer's protocols.^39^

### 2.10 IMQ‐induced psoriasis‐like mouse model

Female C57BL6 mice (18–20 g, 6–8 weeks old) were purchased from Jinan Pengyue experimental animal breeding Co., Ltd (China). All experiments were approved by the Laboratory Animal Ethical Committees of our hospital. A table of random numbers was used to generate the randomization. Mice were randomly divided into Control, Model, Model+MTX, and Model+Etanercept groups (*n* = 8 per group). As described previously, a daily topical dose of 62.5 mg of IMQ cream (Sichuan Mingxin Pharmaceutical Co., Ltd., China) was used on the skin on both ears of the mouse for eight consecutive days to induce psoriasis‐like mouse model.^40^, ^41^ Mice in the control group were treated with vehicle cream (Vaseline Lanette cream; Fagron, Waregem, Belgium) in the same way. After 8 days of IMQ (or vehicle cream) administration, mice in the Model+MTX were intraperitoneally injected with methotrexate (MTX, 1 mg/kg/day; Beijing Solarbio Science & Technology Co., Ltd., China) for 12 days, and mice in the Model+Etanercept group were intraperitoneally injected with etanercept (4 mg/kg, once every three days) for 12 days.^42^, ^43^ MTX and Etanercept were dissolved in pure water. At the end of treatment, mice were sacrificed. Skin samples of four mice were used to perform hematoxylin and eosin (H&E) staining, and skin samples of four mice were used to perform RT‐qPCR and western blot assay.

### 2.11 H&E staining

The skin of mice was collected and fixed in 10% neutral‐buffered formalin (Sigma‐Aldrich). Next, a graded series (70%, 80%, 95%, and 100%) of alcohol solutions were used to dehydrate, and then the samples were embedded in paraffin. Subsequently, the tissue samples were sliced into 5 μm sections, and the sections were dewaxed with xylene and subjected to gradient dehydration. Finally, the sections were stained with hematoxylin and eosin (Merck KGaA, Darmstadt, Germany), and pathological changes were evaluated under a light microscope (Zeiss, Germany).^44^ Image‐Pro Plus software (NIH) was made by use of analyzing the epidermal thickness.

### 2.12 Statistical analysis

GraphPad Prism7.0 software (USA) and SPSS software were used for statistical analysis, and all data were presented as the mean ± standard deviation from three independent experiments. Statistical significance was analyzed using a one‐way analysis of variance followed by Tukey's post hoc test. *p*‐Values less than 0.05 were considered statistically significant.

## 3 RESULTS

### 3.1 Etanercept inhibits proliferation and induces cell cycle arrest and apoptosis in HaCaT cells

**FIGURE 1:**
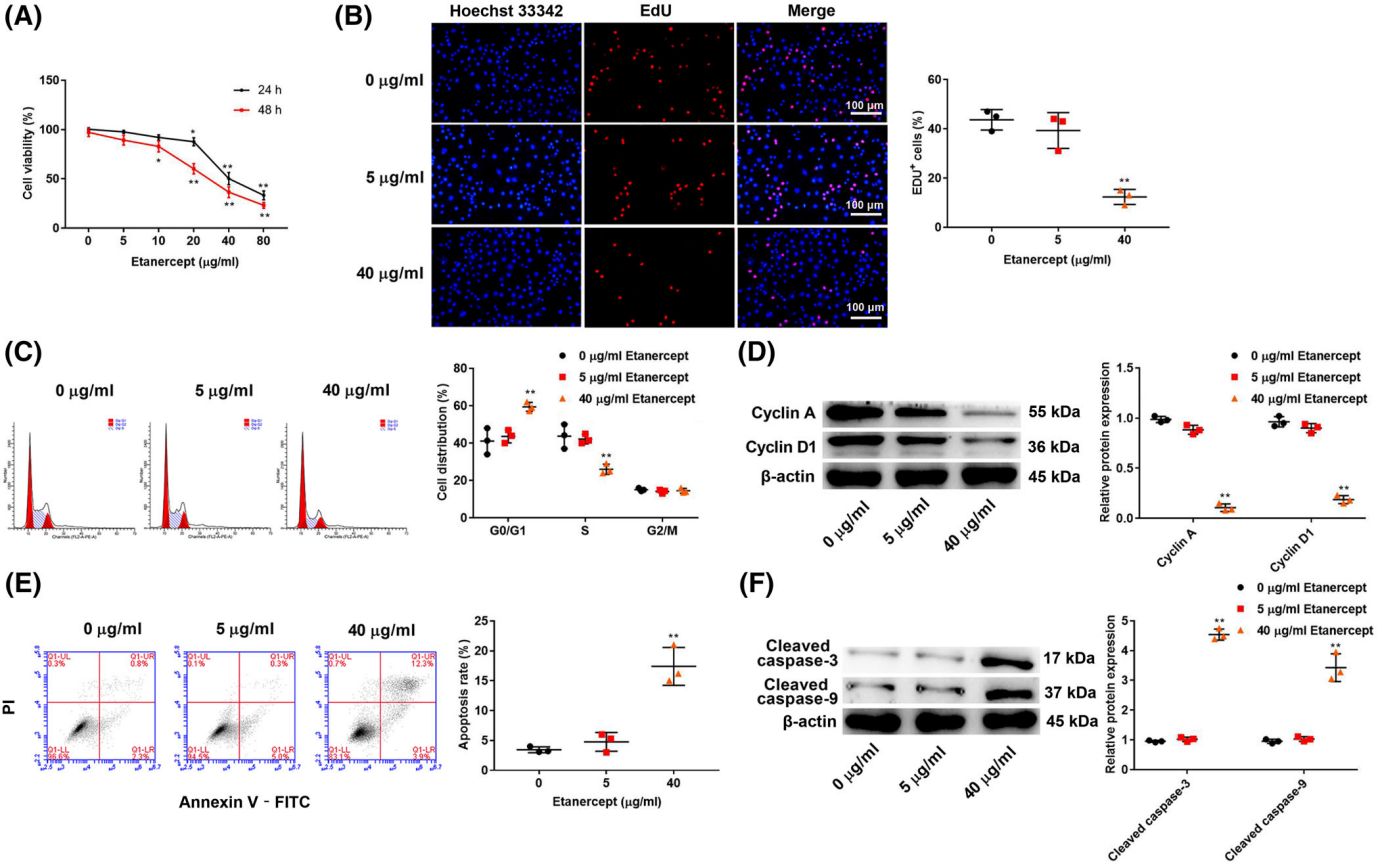
Etanercept inhibited proliferation and induced cell cycle arrest and apoptosis in HaCaT cells. (A) Cell viability of HaCaT cells was detected by Cell Counting Kit‐8 (CCK‐8) assay. (B) 5‐ethynyl‐2′‐deoxyuridine (EdU) assay was carried out to detect the proliferation ability of HaCaT cells. (C) The cell cycle of HaCaT cells was analyzed by flow cytometry. (D) The protein expression of Cyclin A and Cyclin D1 in HaCaT cells was detected by western blot analysis. (E) Cell apoptosis of HaCaT cells was analyzed by flow cytometry. (F) The protein expression of cleaved caspases‐9 and cleaved caspases‐3 was detected by western blot analysis. *N* = 3, data are representative of three experiments. Compared with 0 μg/ml group, *p < 0.05, **p < 0.01.

To detect the effect of etanercept on cell viability of HaCaT cells, cells were treated with different doses of etanercept (0, 5, 10, 20, 40, and 80 μg/ml) for 24 and 48 h. Cell viability was detected by CCK‐8 assay. As shown in Figure 1A, etanercept treatment significantly inhibited cell viability of HaCaT cells in a dose‐ and time‐dependent manner (*p* < 0.05). Based on the above results, etanercept at 5 and 40 μg/ml that had no significant and significant toxic effect on cell viability were chosen for further experiments. Next, an EdU assay was carried out to verify the proliferation ability. The results showed that treatment of 40 μg/ml etanercept reduced EdU‐positive cells compared to 0 μg/ml etanercept (*p* < 0.01), while 5 μg/ml etanercept showed no obvious changes (*p* > 0.05, Figure 1B). Moreover, cell cycle analysis showed that treatment of 40 μg/ml etanercept increased the G0/G1 stage of HaCaT cells and decreased the S stage of HaCaT cells compared to 0 μg/ml etanercept (*p* < 0.01), while 5 μg/ml etanercept showed no obvious changes (*p* > 0.05, Figure 1C). Consistently, treatment of 40 μg/ml etanercept reduced the protein expression of Cyclin A and Cyclin D1 (*p* < 0.01, Figure 1D). In addition, to determine whether the growth inhibitory effect of etanercept was associated with apoptosis, flow cytometry was performed to evaluate the effect of etanercept on HaCaT cell apoptosis. The results showed that treatment of 40 μg/ml etanercept increased the percentage of apoptosis cells (*p* < 0.01, Figure 1E). We then measured the expression of apoptosis‐related proteins (cleaved caspases‐9 and cleaved caspases‐3) using western blot analysis. As shown in Figure 1F, treatment of 40 μg/ml etanercept increased the protein expression of cleaved caspases‐9 and cleaved caspases‐3 (*p* < 0.01), while 5 μg/ml etanercept showed no obvious changes (*p* > 0.05).

### 3.2 Etanercept inhibits proliferation and promotes cell cycle arrest and apoptosis in LPS‐induced HaCaT cells

**FIGURE 2:**
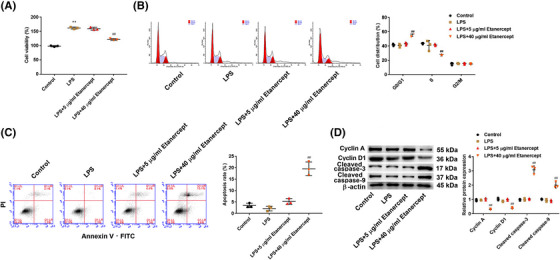
Etanercept inhibited proliferation and promoted cell cycle arrest and apoptosis in lipopolysaccharide (LPS)‐induced HaCaT cells. (A) Cell viability of HaCaT cells was detected by Cell Counting Kit‐8 (CCK‐8) assay. (B) The cell cycle of HaCaT cells was analyzed by flow cytometry. (C) Cell apoptosis of HaCaT cells was analyzed by flow cytometry. (D) The protein expression of Cyclin A, Cyclin D1, cleaved caspases‐9, and cleaved caspases‐3 in HaCaT cells was detected by western blot analysis. *N* = 3, data are representative of three experiments. Compared with control group, **p < 0.01; compared with LPS group, ^##^ *p* < 0.01.

To investigate the effects of etanercept in psoriasis, we stimulated HaCaT cells with LPS to generate cellular psoriatic changes. As shown in Figure 2A, HaCaT cells treated with LPS exhibited a significantly increased rate of cell proliferation compared with the control group (*p* < 0.01). Besides, 40 μg/ml etanercept inhibited proliferation and promoted cell cycle arrest and apoptosis in LPS‐induced HaCaT cells (*p* < 0.01), while 5 μg/ml etanercept showed no obvious changes (*p* > 0.05, Figure 2A–C). Furthermore, under LPS stimulation, the protein expression of Cyclin A and Cyclin D1 in HaCaT cells treated with 40 μg/ml etanercept was downregulated (*p* < 0.01, Figure 2D), and the protein expression of cleaved caspases‐9 and cleaved caspases‐3 was upregulated in 40 μg/ml etanercept treated cells (*p* < 0.01, Figure 2D).

### 3.3 Etanercept decreases the production of inflammatory cytokines and inhibits the HMGB1 signaling pathway in LPS‐induced HaCaT cells

**FIGURE 3:**
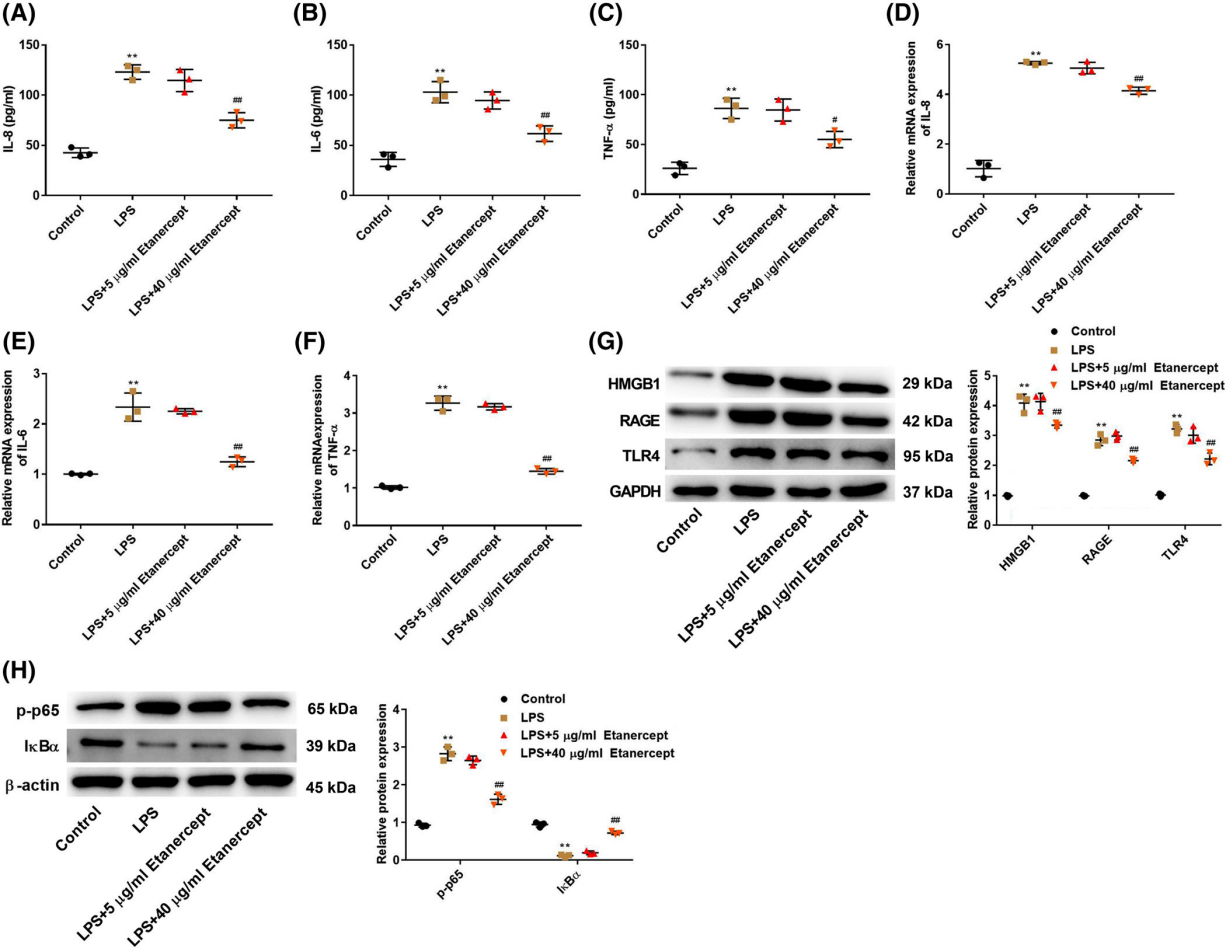
Etanercept decreased the production of inflammatory cytokines and inhibited the high mobility group box 1 (HMGB1) signaling pathway in lipopolysaccharide (LPS)‐induced HaCaT cells. The level of interleukin (IL)‐8 (A), IL‐6 (B), and tumor necrosis factor‐alpha (TNF‐α) (C) in HaCaT cells. The mRNA expression of IL‐8 (D), IL‐6 (E), and TNF‐α (F) in HaCaT cells was measured using reverse transcription‐quantitative polymerase chain reaction (RT‐qPCR) assay. (G) The protein expression of HMGB1, receptor for advanced glycation end products (RAGE), and toll‐like receptor 4 (TLR4) in HaCaT cells was detected by western blot analysis. (H) The protein expression of p‐p65 and IκBα in HaCaT cells was detected by western blot analysis. *N* = 3, data are representative of three experiments. Compared with control group, **p < 0.01; compared with LPS group, ^##^ *p* < 0.01 and ^#^ *p* < 0.05.

The effect of etanercept on the inflammatory cytokines (IL‐8, IL‐6, and TNF‐α) in LPS‐induced HaCaT cells were measured using commercial ELISA kits and RT‐qPCR assay. As shown in Figure 3A–C, LPS treatment increased the levels of IL‐8, IL‐6, and TNF‐α (*p* < 0.01), while 40 μg/ml etanercept markedly attenuated these changes induced by LPS (*p* < 0.01). Meanwhile, the trend of RT‐qPCR results was consistent with that of ELISA (Figure 3D–F). In addition, HMGB1 signaling pathway in HaCaT cells after LPS and/or etanercept treatment was detected by western blot analysis. The results showed that LPS treatment increased the protein expression of HMGB1, RAGE, and TLR4 (*p* < 0.01, Figure 3G), while 40 μg/ml etanercept notably attenuated the LPS‐induced activation of the HMGB1 signaling pathway. The NF‐κB signaling pathway is influenced by HMGB1, we next detected the change in the NF‐κB signaling pathway. As shown in Figure 3H, LPS increased p‐p65 expression and decreased IκBα expression (*p* < 0.01), but 40 μg/ml etanercept reversed these changes induced by LPS.

### 3.4 Etanercept suppresses proliferation and inflammation triggered by LPS in HaCaT cells via the HMGB1 signaling pathway

**FIGURE 4:**
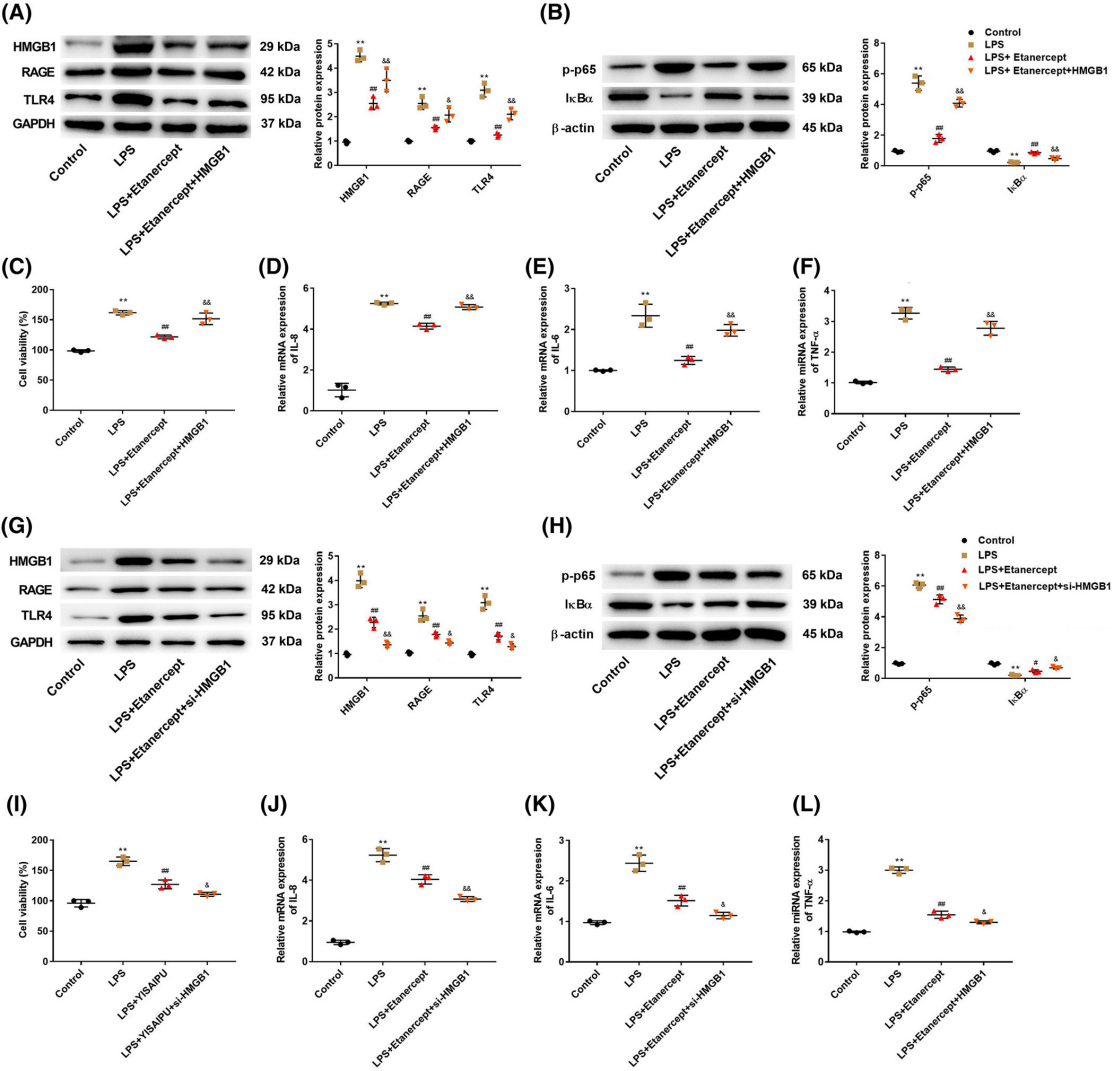
Etanercept suppressed proliferation and inflammation triggered by lipopolysaccharide (LPS) in HaCaT cells via the high mobility group box 1 (HMGB1) signaling pathway. (A and G) The protein expression of HMGB1, receptor for advanced glycation end products (RAGE), and toll‐like receptor 4 (TLR4) in HaCaT cells was detected by western blot analysis. (B and H) The protein expression of p‐p65 and IκBα in HaCaT cells was detected by western blot analysis. (C and I) Cell viability of HaCaT cells was detected by Cell Counting Kit‐8 (CCK‐8) assay. The mRNA expression of interleukin (IL)‐8 (D and J), IL‐6 (E and J), and tumor necrosis factor‐alpha (TNF‐α) (F and L) in HaCaT cells was measured using reverse transcription‐quantitative polymerase chain reaction (RT‐qPCR) assay. *N* = 3, data are representative of three experiments. Compared with control group, **p < 0.01; compared with LPS group, ^##^ *p* < 0.01; compared with LPS+Etanercept group, ^&^ *p* < 0.05 and ^&&^ *p* < 0.01.

In order to investigate whether the effects of etanercept were associated with the HMGB1 signaling pathway, HMGB1 was overexpressed or low expressed in LPS‐induced HaCaT cells. The results of western blot analysis revealed that pcDNA3.1‐HMGB1 transfection markedly increased the expression of HMGB1, RAGE, and TLR4 compared with the LPS+Etanercept group (*p* < 0.01), while siRNA‐HMGB1 transfection further decreased the expression of HMGB1, RAGE, and TLR4 compared with LPS+Etanercept group (*p* < 0.05, Figure 4A,G). Moreover, compared with the LPS+Etanercept group, overexpression of HMGB1 increased p‐p65 expression and decreased IκBα expression (*p* < 0.01), while knockdown of HMGB1 further decreased p‐p65 expression and increased IκBα expression (*p* < 0.05, Figure 4B,H). Figure 4C displayed that overexpression of HMGB1 notably reversed the inhibitory effects of etanercept on LPS‐induced HaCaT cell viability (*p* < 0.01), while knockdown of HMGB1 reduced HaCaT cell viability compared with LPS+Etanercept group (*p* < 0.05, Figure 4I). In addition, compared with the LPS+Etanercept group, the mRNA expression of IL‐8, IL‐6, and TNF‐α was also increased in the LPS+Etanercept+HMGB1 group (*p* < 0.01, Figure 4D–F). Compared with the LPS+Etanercept group, the knockdown of HMGB1 reduced the mRNA expression of IL‐8, IL‐6, and TNF‐α (*p* < 0.05, Figure 4J–L).

### 3.5 Etanercept ameliorates inflammation and inhibits HMGB1 signaling pathway in an IMQ‐induced psoriasis‐like mouse model

**FIGURE 5:**
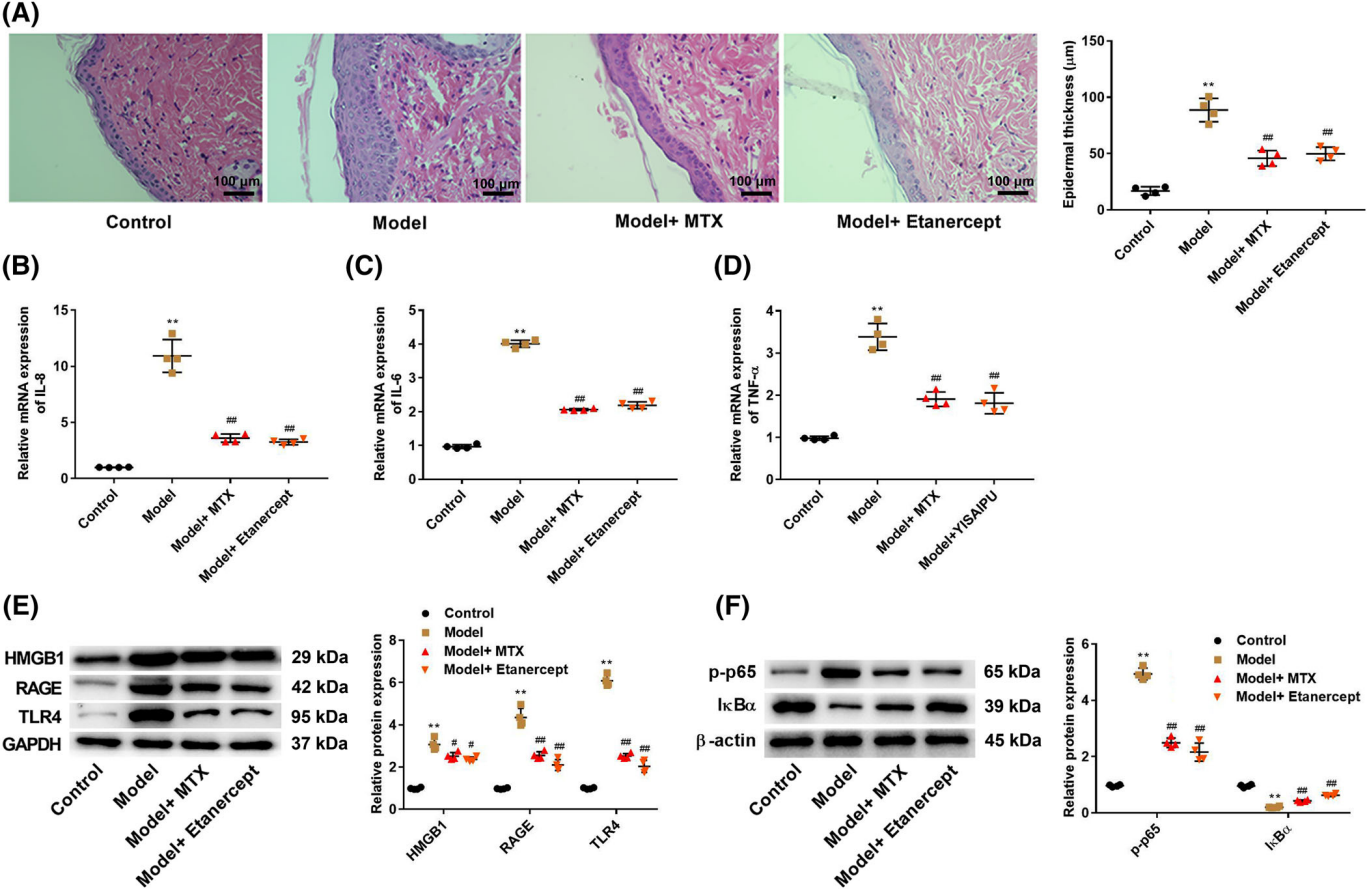
Etanercept ameliorated inflammation and inhibited high mobility group box 1 (HMGB1) signaling pathway in an imiquimod (IMQ)‐induced psoriasis‐like mouse model. (A) The histopathology of the mouse ear tissue was detected using hematoxylin and eosin staining. The mRNA expression of interleukin (IL)‐8 (B), IL‐6 (C), and tumor necrosis factor‐alpha (TNF‐α) (D) in the skin of mice was measured using reverse transcription‐quantitative polymerase chain reaction (RT‐qPCR) assay. (E) The protein expression of HMGB1, receptor for advanced glycation end products (RAGE), and toll‐like receptor 4 (TLR4) in the skin of mice was detected by western blot analysis. (F) The protein expression of p‐p65 and IκBα in the skin of mice was detected by western blot analysis. *N* = 4, data are representative of three experiments. Compared with control group, **p < 0.01; compared with model group, ^#^ *p* < 0.05 and ^##^ *p* < 0.01.

To further analyze the anti‐psoriatic effect of etanercept in vivo, we established an IMQ‐induced psoriasis‐like mouse model. As shown in Figure 5A, IMQ increased the epidermal thickness, induced the formation of rete ridges, and was accompanied by inflammatory cell infiltration, while MTX (as a reference agent) and etanercept reduced epidermal thickening and inhibited the formation of rete ridges and inflammatory cell infiltration induced by IMQ. In addition, the mRNA expression of IL‐8, IL‐6, and TNF‐α was measured using RT‐PCR assay. The results showed that the mRNA expression of IL‐8, IL‐6, and TNF‐α was significantly higher in IMQ‐induced mice than in those from control mice (*p* < 0.01), while MTX and etanercept treatment decreased the mRNA expression of IL‐8, IL‐6, and TNF‐α (*p* < 0.01, Figure 5B–D). To investigate the role of etanercept on the HMGB1 signaling pathway and NF‐κB signaling pathway, the protein expression of HMGB1, RAGE, TLR4, p‐P65, and IκBα in IMQ‐induced psoriasis‐like skin lesions was detected by western blot analysis. As shown in Figure 5E,F, compared with the control group, IMQ increased the protein expression of HMGB1, RAGE, TLR4, and p‐P65 and reduced IκBα (*p* < 0.01), after treatment with MTX and etanercept, the protein expression of HMGB1, RAGE, TLR4, and p‐P65 was decreased, IκBα expression was enhanced (*p* < 0.01).

## 4 DISCUSSION

Psoriasis is a common immune‐mediated skin disease, which causes complications (such as kidney dysfunction and hepatic inflammation) and brings great damage to the quality of patients’ life.^41^, ^45^, ^46^, ^47^ The pathogenesis of psoriasis is involved in the hyperproliferation of keratinocytes and infiltration of immune cells (such as T cells, neutrophils, and macrophages) in the skin.^48^ In the present study, the aim was to investigate the role and related mechanisms of etanercept in the psoriasis model in vitro and in vivo. We found that etanercept inhibited proliferation and inflammation, promoted cell cycle arrest and apoptosis in LPS‐induced HaCaT cells, and etanercept ameliorated inflammation in an IMQ‐induced psoriasis‐like mouse model. Moreover, the therapeutic effects of etanercept in psoriasis were related to the HMGB1 signaling pathway.

Epidermal keratinocyte hyperproliferation and poor differentiation are the most obvious characteristics of psoriasis.^49^ Therefore, it is important to inhibit the abnormal proliferation of keratinocytes. In this research, our results revealed that treatment of etanercept inhibited cell viability of HaCaT cells in a dose‐ and time‐dependent manner. To investigate the effects of etanercept in psoriasis, HaCaT cells were treated with LPS to generate cellular psoriatic changes. We found that LPS treatment increased cell viability compared with the control group, while etanercept significantly inhibited cell viability in psoriasis models in vitro. According to a previous study, the cell cycle can affect cell proliferation.^50^ Subsequently, we assessed the cell cycle and the expression of cell cycle‐related proteins (Cyclin A and Cyclin D1). Cyclin D1 has a role in the cell cycle of the G1 to S phase, while cyclin A is a regulator in the transition of the G2 to the M phase.^51^, ^52^, ^53^ In addition, previous studies suggested that cyclin A protein expression in the epidermis was enhanced in psoriasis, and the upregulation of cyclin D1 was correlated with the hyperproliferation of the epidermis.^54^, ^55^ Our results showed that etanercept promoted G1 phase arrest by decreasing the expression of cyclin A and cyclin D1. In addition, it has been reported that cell apoptosis plays an important role in the pathogenesis of skin diseases, and diminished keratinocyte apoptosis has been found in psoriatic lesions.^56^ Thus, the drugs inducing keratinocyte apoptosis may have the potential to treat skin diseases. We then analyzed the effect of etanercept on HaCaT cell apoptosis and found that etanercept induced cell apoptosis and increased the protein expression of cleaved caspases‐9 and cleaved caspases‐3 in LPS‐induced HaCaT cells. These findings demonstrated that etanercept might exert a therapeutic effect by inhibiting proliferation and promoting apoptosis of keratinocytes.

Cutaneous inflammation is another characteristic of psoriasis.^57^ Keratinocytes are the first barrier against external environmental threats, and it was reported that hyperplastic keratinocytes react to a variety of cytokines in the lesion, and then produce pro‐inflammatory cytokines to aggravate inflammatory response, thus forming a positive feedback loop.^58^, ^59^, ^60^, ^61^ Therefore, it is possible that the anti‐psoriasis effect of etanercept may depend on the regulation of some cytokines. TNF‐α, IL‐8, and IL‐6 are commonly expressed in patients with psoriasis.^57^, ^62^, ^63^ Previous studies have reported that TNF‐α is a main cytokine related to the pathogenesis of psoriasis, and it participates in the regulation of keratinocyte proliferation, activation of immune cells, and production of various pro‐inflammatory mediators.^64^, ^65^, ^66^ IL‐6 is considered to be a major inducer of the regulated expression of many cytokines.^67^ As a component of normal human skin, IL‐6 stimulates keratinocyte proliferation and suppresses terminal keratinocyte differentiation, which results in hyperkeratosis and parakeratosis in psoriasis.^57^, ^67^ Furthermore, IL‐6 and TNF‐α have been reported to promote and increase the severity of epidermal inflammation.^68^ IL‐8, a common chemokine, is involved in new vessel formation and keratinocyte proliferation ^69^. The activated keratinocytes can produce higher levels of IL‐8 in psoriasis vulgaris, and the local level of IL‐8 is positively correlated with the severity of the disease.^70^, ^71^ In this study, the results of ELISA and RT‐qPCR demonstrated that etanercept decreased the production of inflammatory cytokines (IL‐8, IL‐6, and TNF‐α) in LPS‐induced HaCaT cells. To analyze the effect of etanercept on psoriasis in vivo, a psoriasis‐like mouse model induced by IMQ was established to simulate the disease. MTX has a therapeutic effect on psoriasis.^72^ Our findings suggested that etanercept decreased the expression of IL‐8, IL‐6, and TNF‐α, and alleviate IMQ‐induced pathological changes. Moreover, the therapeutic effect of etanercept on mice was similar to that of MTX. Taken together, these findings demonstrated that etanercept exerted a therapeutic effect by inhibiting inflammation.

HMGB1 is a widely expressed multifunctional inflammatory factor, which is involved in various biological processes, such as tissue remodeling, embryonic development, and tumorigenesis.^73^, ^74^ Previous study has demonstrated that HMGB1 levels were increased in the dermis of psoriatic skin, which indicates that HMGB1 may be related to the pathogenesis of psoriasis.^75^ Recent studies have demonstrated that HMGB1 can regulate RAGE and TLR4 receptors, and the HMGB1 pathway contributes to inflammation via multiple mechanisms.^76^, ^77^ In the immune response, NF‐κB is a key downstream signal pathway of TLR regulation.^78^ The activation of the NF‐κB pathway causes the release of proinflammatory cytokines, such as TNF‐α and IL‐1β.^79^ Therefore, the inhibition of the HMGB1 signaling pathway may effectively regulate the progression of psoriasis. Based on this hypothesis, we evaluated the protein expression of HMGB1, RAGE, TLR4, p‐P65, and IκBα in tissues and HaCaT cells. Our results showed that etanercept decreased the protein expression of HMGB1, RAGE, TLR4, and p‐p65 and increased IκBα expression compared with cell and animal models. Moreover, the results of in vitro experiments showed that overexpression of HMGB1 increased HaCaT cell viability and inflammation compared with the LPS+etanercept group, while knockdown of HMGB1 further enhanced the inhibitory effects of etanercept on LPS‐induced HaCaT cell viability and inflammation. Taken together, these findings indicated that etanercept exerted anti‐inflammatory and protective effects in LPS‐induced HaCaT cells through the inactivation of the HMGB1 signaling pathway.

However, there is a limitation in the study. The animal study was only divided into control, Model, Model+MTX, and Model+Etanercept group. In order to make the experiment more complete, we should add the Normal group (healthy mice without any treatment) and the Model+vehicle group.

## 5 CONCLUSIONS

Overall, etanercept ameliorated psoriasis progression, as evidenced by inhibiting proliferation and inflammation, and promoted cell cycle arrest and apoptosis in keratinocytes. Moreover, the mechanism of etanercept might be related to the inactivation of the HMGB1 signaling pathway in the treatment of psoriasis. Our findings may provide an experimental basis for understanding the mechanism of etanercept in psoriasis.

## AUTHOR CONTRIBUTIONS

Shu Li designed the study; Guangli Li and Xiaoyan Li performed the research; Fan Wu, Ling Li, and Shu Li analyzed the data and wrote the paper.

## CONFLICT OF INTEREST STATEMENT

The authors declare no conflict of interest.

## FUNDING INFORMATION

No funding.

## ETHICS STATEMENT

The experimental protocol of our study was performed in accordance with the Guide for the Care and Use of Laboratory Animals and approved by Taizhou People's Hospital.

## Data Availability

The datasets used and analyzed during the current study are available from the corresponding author upon reasonable request.
